# Suggestive answers strategy in human-chatbot interaction: a route to engaged critical decision making

**DOI:** 10.3389/fpsyg.2024.1382234

**Published:** 2024-03-28

**Authors:** Yusuke Yamamoto

**Affiliations:** School of Data Science, Nagoya City University, Nagoya, Japan

**Keywords:** human-AI interaction, large language model, behavior change, critical information-seeking, chatbot

## Abstract

In this study, we proposed a novel chatbot interaction strategy based on the *suggestive ending* of answers. This strategy is inspired by the cliffhanger ending narrative technique, which ends a story without specifying conclusions to spark readers' curiosity as to what will happen next and is often used in television series. Common chatbots provide relevant and comprehensive answers to users' questions. In contrast, chatbots with our proposed strategy end their answers with hints potentially interest-triggering users. The suggestive ending strategy aims to stimulate users' inquisition for critical decision-making, relating to a psychological phenomenon where humans are often urged to finish the uncompleted tasks they have initiated. We demonstrated the implication of our strategy by conducting an online user study involving 300 participants, where they used chatbots to perform three decision-making tasks. We adopted a between-subjects factorial experimental design and compared between the following UIs: (1) *plain* chatbot—it provides a generated answer when participants issue a question; (2) *expositive* chatbot—it provides a generated answer for a question, adding short summaries of a positive and negative person's opinion for the answer; (3) *suggestive* chatbot—it provides a generated answer for a question, which ends with a suggestion of a positive and negative person for the answer. We found that users of the *suggestive* chatbot were inclined to ask more questions to the bot, engage in prolonged decision-making and information-seeking actions, and formulate their opinions from various perspectives. These findings vary with the users' experience with *plain* and *expositive* chatbots.

## 1 Introduction

Recent advancements in artificial intelligence (AI), particularly the remarkable evolution of large language models (LLMs), have given rise to a lot of services and applications that support human tasks in various domains. Generative AI with LLMs holds a strong potential for substantially changing human intellectual activities. For example, instruction-tuned LLMs (e.g., ChatGPT) can quickly generate surprisingly natural sentences in response to human questions (Wei et al., 2021). Zylowski and Wölfel (2023) revealed that when specifying personas for ChatGPT in prompts enables it to simulate a variety of personalities and capabilities. OpenAI reported that ChatGPT scored 1,300/1,600 on the SAT^1^ by eliciting knowledge in its language model^2^. In 2024, Google released *Gemini Ultra*, the highly capable LLM which outperforms GPT-4 on text-based tasks, including reasoning, reading comprehension, and code generation (Team et al., 2023). Furthermore, an appropriate understanding of LLM applications and their effective use can equally support decision-making and opinion formulation (Wambsganss et al., 2020, 2021; Jakesch et al., 2023; Petridis et al., 2023).

Despite their superlative functionalities, generative AIs with LLMs often generate incorrect, biased, or unrealistic information, a phenomenon known as *hallucination* (Maynez et al., 2020). Overreliance on AIs causes automation bias to users (Goddard et al., 2011), leading to the ubiquitous obliviousness of AI-generated false information (Lakkaraju and Bastani, 2020). Studies have shown that overusing AIs can inhibit the development of users' cognitive skills (Noyes, 2007; Carr, 2014), naturally affecting their critical thinking abilities. As a result, users can be unconsciously led to a specific polarity by opinionated AI assistants for writing (Jakesch et al., 2023). These aspects raise serious educational concerns. For instance, students using generative AI-powered chatbots can accept harmful/incorrect information without doubt, which strongly affects the development of their critical thinking and problem-solving skills (Kasneci et al., 2023).

Although the research on improving the performance of generative AIs with LLMs is under extensive development, undesirable output information remains highly probable (Wei et al., 2021; Nakano et al., 2022; Tay et al., 2022; Wang et al., 2023). This probability is particularly aggravated by the human *confirmation bias*, defined as the tendency to preferentially view or search for information consistent with one's opinions or hypotheses (Kahneman, 2011). Therefore, improving generative AIs should be accompanied by an effective design of human–AI interactions that promote users' cognitive activities for critical decision-making or opinion formulation.

**Figure 1:**
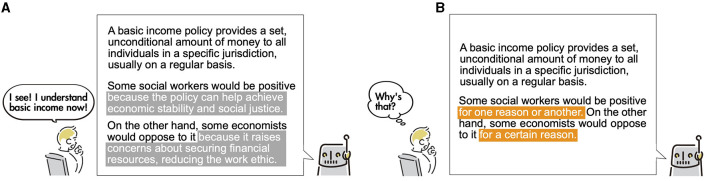
Comparison between *expositive* chatbot and *suggestive* chatbot. **(A)** Expositive chatbots provide relevant answers to questions and brief supplementary information on a specific perspective. **(B)** Suggestive chatbots (the proposed bot) provide answers ending with suggestive messages hinting at something. Suggestive chatbots encourage users to ask spontaneous questions for proactive critical decision-making.

In this study, we proposed a novel human–chatbot interaction strategy, *suggestive ending*, for generative AI-powered chatbot answers to foster decision-making from various perspectives. Our method is inspired by the *cliffhanger ending* narrative technique, which ends a story without specifying conclusions to spark readers' curiosity as to what will happen next. The cliffhanger method is often used in television series. It relates to a psychological phenomenon known as the *Ovsiankina effect*, where humans are often urged to finish the uncompleted tasks they have initiated (Wirz et al., 2023). *Suggestive* bots employed with the proposed strategy output their answers with hints to potentially interest-triggering subjects (Figure 1B). In contrast, common chatbots provide relevant and comprehensive answers to users' questions (Figure 1A). Therefore, when interacting with suggestive chatbots on a given theme, users' proactive critical decision-making is stimulated by intentionally leaving room for questions.

We conducted an online user study involving 300 participants to validate our proposed method on the human–AI interaction. Results revealed the following three primary observations.

- When using the suggestive bot, participants engaged in decision-making by inputting questions to the bot. This has led participants to spend longer interactions with the suggestive bot than the plain (i.e., simply providing relevant answers) and expositive (i.e., providing relevant answers with supplementary information) bots.
- Compared to the plain bot, participants were likely to write longer opinions from various perspectives using the suggestive bot.
- When using either the expositive or plain bot, participants showed similar efforts in their decision-making activities.

## 2 Related work

### 2.1 AI-assisted decision-making and opinion formation

AI systems developed to assist decision-making and opinion formation have been studied from the viewpoints of supporting interpretation of AI predictions, improving the understanding of arguments, enhancing the efficiency of opinion formulation, searching for supportive information, etc.

It is essential to understand how and why black-box AIs provide predictions for users to efficiently use them during decision-making. Hence, many researchers have studied *explainable AI* technologies to improve the interpretability of machine learning (ML) models. For instance, for ML behaviors on structured data, researchers have proposed various methods to summarize the contributed features to predictions (Lundberg and Lee, 2017; Fisher et al., 2019) and explain how the models work with data examples (Kim et al., 2016). Lakkaraju and Bastani (2020) reported misleading explanations on black-box MLs as a cause for users trusting even harmful MLs. Therefore, considering the characteristics of human design thinking is important to improve the interpretability of AIs for decision-making.

To better understand the aforementioned arguments, Wambsganss et al. (2021) proposed ArgueTutor, a chatbot system that provides users with feedback to identify sentences in their documents that require logical improvement. Furthermore, they proposed an interactive system to visualize the argumentation structure of a given document, thereby helping users make more logical judgments (Wambsganss et al., 2020). Petridis et al. (2023) developed AngleKindling, a system that supports journalists in exploring points to scrutinize potential negative impacts on press releases using an LLM.

Several investigations have been conducted on *suggestive keyboards* to support efficient opinion formulation. Arnold et al. (2016) proposed a phrase-suggesting method for text composition instead of predicting words following users' input texts. However, *suggestive keyboard* technologies could affect what to write. In another study, Arnold et al. (2020) reported that *suggestive keyboard* technologies affect the writers' choices who often follow AI-based text suggestions, while it improves their writing speed. Jakesch et al. (2023) reported that when suggestive keyboards were used, in which an LLM was fine-tuned to suggest positive (negative) phrases, users were likely to write positive (negative) opinions.

In the field of information retrieval (IR), several researchers have investigated systems for searching information to support decision-making. Rinott et al. (2015) proposed a method to search for evidence supporting a given claim from unstructured documents. Liu et al. (2022) proposed Crystalline, a browser developed to tabulate collected Web information for efficient decision-making.

The aforementioned studies reveal that users with sufficient skills and motivation to properly use advanced AI technologies can obtain useful assistance from these technologies in decision-making and opinion formulation. Otherwise, overreliance on AIs for decision-making causes negative impacts on users, including shortsighted decision making, cognitive downskilling, and opinion radicalization. Therefore, our proposed method focuses on eliciting questions from chatbot users and promoting active opinion formation in the human–chatbot interaction.

### 2.2 Generative information retrieval

With the emergence of LLMs, changes were introduced to the conventional IR model, which aims to provide a ranked list of relevant documents for a keyword query. *Generative IR* is a new LLMs-based paradigm of generating information to directly answer users' questions. When a question is given, typical generative IR systems (i.e., AI-powered chatbots) extend prompts with likely completions and extract answers from the extended prompts (Najork, 2023).

ChatGPT^3^ and Google Gemini^4^ are recently developed generative IR applications that have spurred unprecedented universal attention. Nevertheless, ongoing research is highlighting their drawbacks, such as generating incorrect or unrealistic answers, which is known as the *hallucination* phenomenon (Maynez et al., 2020). Metzler et al. (2021) reported several challenges in generative IRs, such as suggesting contexts for generated answers and considering the authority or quality of documents for answer generation. Several methods have been proposed to tackle these challenges, such as tuning LLM models for human-favorable answers (Wei et al., 2021), linking generated answers (or questions) with relevant documents (Nakano et al., 2022; Tay et al., 2022), and improving the interpretability of generative AIs (Sun et al., 2022). Furthermore, Wang et al. (2023) proposed *Shepherd*, an LLM model that provides feedback to improve target LLMs by analyzing the generated texts.

While the aforementioned studies focus on the performance improvement and high functionality of generative IR systems, our proposed method focuses on enhancing users' information-seeking and cognitive activities in generative IRs.

### 2.3 Enhancing critical information seeking and decision-making

Studies conducted to activate and enhance information-seeking and decision-making abilities can be categorized into two approaches for steering and empowering better judgments: *nudging* (Thaler and Sunstein, 2009; Caraban et al., 2019) and *boosting* (Hertwig and Grne-Yanoff, 2017).

*Nudging* is defined as “*an approach to alter people's behavior in a predictable way without forbidding any option or substantially changing their economic incentive”* (Thaler and Sunstein, 2009). In the field of HCI, several methods have been reported for the application of this concept, which include supporting critical information seeking (Yamamoto and Yamamoto, 2018; Saito et al., 2020; Ihoriya et al., 2022; Suzuki and Yamamoto, 2022) and enhancing privacy awareness on the Web (Wang et al., 2013; Zimmerman et al., 2019; Yamamoto and Yamamoto, 2020). For example, Yamamoto and Yamamoto (2018) proposed the query priming system, which inserts queries to evoke critical thinking during query completion/recommendation in a search system. Suzuki and Yamamoto (2022) proposed a search user interface (UI) that makes web searchers reflect on their webpage selection criteria and promote content-quality-oriented web searches regardless of visual appearances. Wang et al. (2013) proposed a privacy nudge that shows Facebook profile pictures of the target audience when users post content on Facebook to enhance users' awareness to potential risks.

Nudging supports better decision-making by focusing on related systematic biases. However, *boosting* is an intervention to improve cognitive competence for proactive and rational decision-making (Hertwig and Grne-Yanoff, 2017). Shimizu et al. (2022) proposed *privacy-aware* snippets, which aim to enhance privacy risk judgment in Web searches by providing comprehensive information about sharing conditions of browsing histories. Harvey et al. (2015) reported that providing examples of high-quality queries can help users learn to improve the efficiency of their query formulation. Buçinca et al. (2021) reported that the users' final decision-making performance can be improved if they are required to think by themselves before the AIs provide supportive information for decision-making. Danry et al. (2023) reported that when AIs ask people a simple question to confirm a claim's logical validity, reasoning activities can be activated, and the fallacy identification performance can be improved.

While questioning approaches such as Danry et al. (2023)'s method are explicit boosting (i.e., instructive intervention), our method is regarded as implicit boosting (i.e., modest intervention). Our proposed strategy aims to trigger users' spontaneous questions through their interaction with chatbots, introducing suggestive messages in answers and leaving room for further questioning. We expect that our suggestive ending approach will be perceived as less intrusive than instructive questioning approaches.

## 3 Research questions

Our proposed *suggestive ending* strategy in chatbots for IR aims to provoke users' questions on a given theme or prior belief, driving them to make theme-dependent critical decisions. Ennis (1987) defined critical thinking as logical and reflective thinking to determine what to believe or do. Furthermore, the author claimed that ideal critical thinkers are disposed to seek reasons, consider entire situations, look for alternatives, and use critical thinking, e.g., deductive reasoning. Several studies revealed the effect of *lateral reading*, a method to check multiple information resources in parallel for critical review on a theme (Meola, 2004; Wineburg and McGrew, 2019; Brodsky et al., 2021). We expect that if chatbots implicitly suggest the existence of things to check at the end of their responses, users would be more willing to critically construct their opinions and gather information for validation compared to cases where chatbots provide detailed explanatory answers.

To explore the validity of our proposed strategy using suggestive bots, we considered the following research questions:

**RQ1**: Do suggestive bots engage users in investing additional effort to form their opinions and gather information for decision-making?
**RQ2**: Do suggestive bots encourage users to consider various perspectives when making their decision?

As we are interested in exploring whether suggestive bots should actively nudge people to question the details of the bot's ambiguous endings, we also investigated the following research question:

**RQ3**: Do question (query) suggestions along with suggestive bot's answer promote more critical decision-making?

According to the elaboration likelihood model theory proposed by Petty and Cacioppo (1986), people often pay more attention to information in which they have sufficient knowledge or strong understanding interest. Otherwise, they often use poor judgment for accepting or rejecting the information. Based on this theory, individual factors can affect people's effort and behavior in decision-making tasks as well as suggestive bot's behaviors. Therefore, we have also formulated the following research question:

**RQ4**: Do individual factors, such as knowledge, interest, and familiarity with the information sought using chatbots, affect associated decision-making tasks?

## 4 Materials and methods

**Figure 2:**
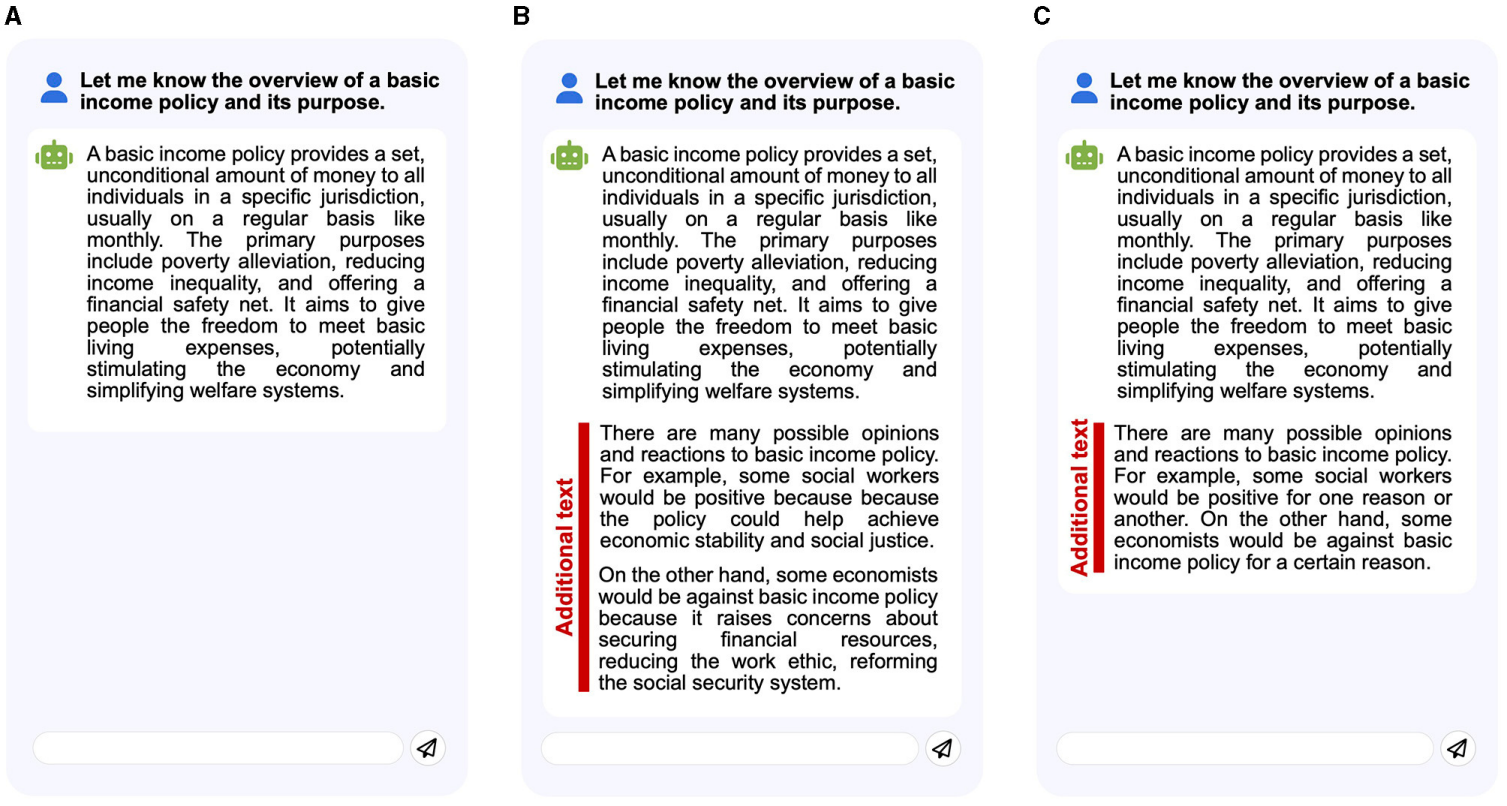
Chatbot user interfaces in our user study. **(A)** plain bot, **(B)** expositive bot, and **(C)** suggestive bot. The differential information between chatbots is represented by red bars (The chatbots did not show these red bars and texts to the participants).

We conducted an online user study to investigate the effect of suggestive ending in AI-powered chatbots on decision-making tasks. The user study was conducted in Japanese (on August 11 and 12, 2023). For this, we adopted a between-subjects factorial experimental design, where the factor is a user interface (UI) condition with four levels:

1. plain chatbot: it provides a generated answer when participants issue a question (query) (Figure 2A).
2. expositive chatbot: provides a generated answer for a question, adding short summaries of a positive and negative person's opinion for the answer (Figure 2B).
3. suggestive chatbot: it provides a generated answer for a question, which ends with a suggestion of a positive and negative person for the answer. Unlike the expositive chatbot, this bot does not show short opinion summaries (Figure 2C).
4. suggestive^++^ chatbot: as an extension of the suggestive chatbot, it provides links to ask about suggested positive/negative people's opinions, following a generated answer (**Figure 4**).

We conducted a user study on a crowdsourcing platform. Crowdsourcing platforms such as Amazon Mechanical Turk^5^ and Prolific^6^ enable researchers to recruit a large number of participants via the internet at lower costs compared to traditional survey companies. Consequently, user studies with crowdsourcing have been becoming popular in the communities of Human-Computer Interaction (HCI) (Kittur et al., 2008; Komarov et al., 2013) and Information Retrieval (IR) (Yamamoto and Yamamoto, 2018; Câmara et al., 2021; Roy et al., 2021) as an alternative way to laboratory-based experiments. Numerous studies have examined the reliability of crowdsourcing by comparing crowd workers' performance to that of participants in laboratory settings (Lutz, 2016; Peer et al., 2017; Hettiachchi et al., 2022). These studies have demonstrated that crowdsourcing can be reliable for conducting user studies, provided that the online tasks are designed to control experimental environments and mitigate satisficing behaviors—whereby participants make judgments or complete tasks with minimal effort. In light of these findings, we conducted a user study with a crowdsourcing service to examine the effectiveness of our proposed method. Note that we implemented an instructional manipulation check (IMC), a popular technique to identify inattentive crowd workers, to ensure the integrity of our data collection process. Furthermore, we rejected crowd workers using mobile/tablet devices so that all participants could perform tasks on the same layout on their PCs.

Participants were randomly allocated into one of the above four UIs. They then conducted tasks to summarize their opinions about three randomly allocated themes. To consider individual differences, we measured and analyzed personal factors as covariates, including the frequency of using chatbots for information seeking, interest in task themes, and familiarity with the themes. We designed this user study following the research ethics guidelines of our affiliated organization.

### 4.1 Themes for decision-making tasks

We prepared eight themes for decision-making tasks and one theme for practice tasks. The themes were prepared from the website of the National Association of Debate in Education, Japan. We selected the frequently used nine themes in debate competitions for high school students in Japan, as listed in Table 1. As presented in Table 1, the impressions of participants indicated their unfamiliarity with most themes on average. Moreover, the interests of participants were slightly inclined to positive polarity on average (excluding *making doggy bags available at restaurants*).

**Table 1:** Themes for decision-making tasks and corresponding participants' impressions.

**Theme**	**Interest**	**Familiarity**	**#Exp. perspectives**
Introduction of daylight saving time	3.77 (1.50)	3.63 (1.44)	5
Introduction of carbon tax	3.77 (1.60)	2.34 (1.37)	5
Charging for ambulance	4.81 (1.23)	2.74 (1.34)	5
Making doggy bags available at restaurants	2.95 (1.64)	1.72 (1.14)	4
Restrictions on whale fishing	3.88 (1.50)	3.22 (1.40)	6
Sales promotion of genome-edited food	3.59 (1.65)	2.05 (1.17)	4
Expanding acceptance of foreign workers	4.73 (1.27)	3.30 (1.38)	6
Restrictions on fake news	4.58 (1.37)	3.37 (1.30)	4
Introduction of universal basic income system (for practice task)	NA	NA	NA

Interest and familiarity use a seven-point scale (1, not at all; 4, neutral; 7, very much). Numbers in the table indicate the mean and standard deviation (in parentheses). #Exp. perspectives mean the number of expected perspectives for each theme.

### 4.2 Chatbots

The aforementioned four UI conditions (chatbots) employed ChatGPT, OpenAI instruction-tuned LLM, via Azure OpenAI Service GPT API (gpt-3.5-turbo^7^) to generate answers for participants' questions. In particular, we used an LLM prompt-engineering technique in the suggestive, suggestive^++^, and expositive bots to complement additional information with plain answers for questions.

One possible way in our proposed *suggestive ending* strategy in chatbots is to suggest perspectives for decision-making explicitly, such as key issues (Câmara et al., 2021; Petridis et al., 2023) and positive/negative aspects for themes (Liao and Fu, 2014; Liao et al., 2015). However, such explicit suggestions are revealing and do not encourage users to proactively reflect on what they should think for their decision-making. On the one hand, studies in the field of learning science indicate that contents should leave proper room for questioning and discussion so that people would be willing to learn a theme and deepen their knowledge (King, 1992). On the other hand, it is difficult for users to find important questions and perspectives for a theme if they lack knowledge and interest.

Therefore, we designed two types of chatbots, namely, (suggestive and suggestive^++^), to provide direct answers to users' questions and additional suggestions on the existence of positive and negative people for a theme, respectively. The two chatbots never suggest the kind of perspectives the positive/negative people can have before users explicitly ask about them.

#### 4.2.1 Suggestive bot

This chatbot suggests examples of positive and negative people for a decision-making theme when the participants ask an *initial question*, an overview of a given theme, and its purpose (Figure 2C). As described in Section 4.3, just after each decision-making task started, we predefined an initial question (query) about an overview of a theme and set it in the query box of the chatbot. When accepting the initial question, the Suggestive bot generated an answer for the question. The bot then suggested an example of a positive and negative persons at the end of the generated answer using the following sentence:

“*There are many possible opinions and reactions to [THEME]. For example, Some [POSITIVE PERSON] would be positive for one reason or another. However, some [NEGATIVE PERSON] would be against [THEME] for a certain reason”*.

**Figure 3:**
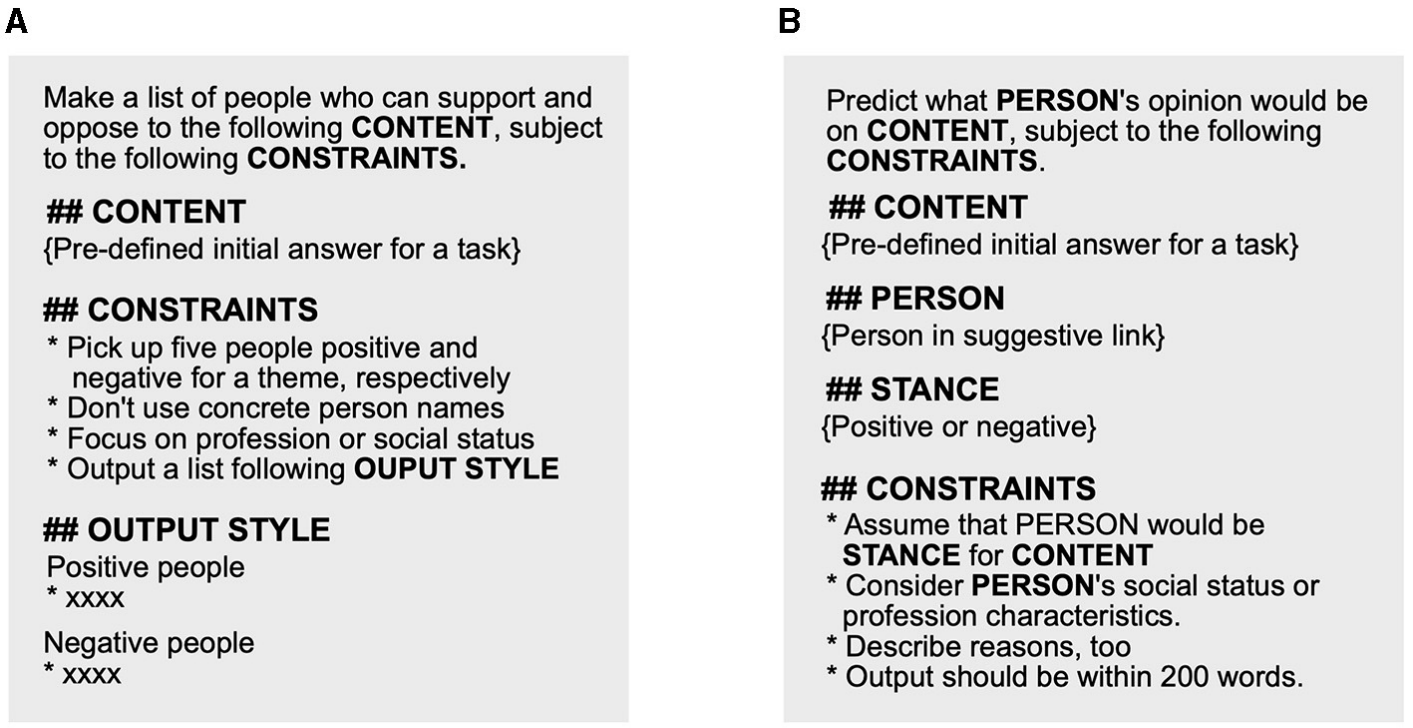
LLM prompts used to generate information for suggestive and suggestive^++^ bots. **(A)** Prompt to gather positive/negative people. **(B)** Prompt to find target person's opinion.

The Suggestive bot finds an example of positive/negative people for a theme as follows:

1. The bot generates an answer (referred to as *initial answer*) for an initial question about a theme by simply fetching Azure OpenAI API with the initial question.
2. The bot gathers a list of people who might have positive/negative feelings for the *initial answer* using the prompt illustrated in Figure 3A.
3. The bot randomly picks up a positive and negative person.

Before the user study, we cached an initial answer and a list of positive/negative people for each theme in Table 1. During the study, the suggestive bot used the cached results for suggestive answer generation so as not to fail due to OpenAI API error.

#### 4.2.2 Suggestive++ bot

**Figure 4:**
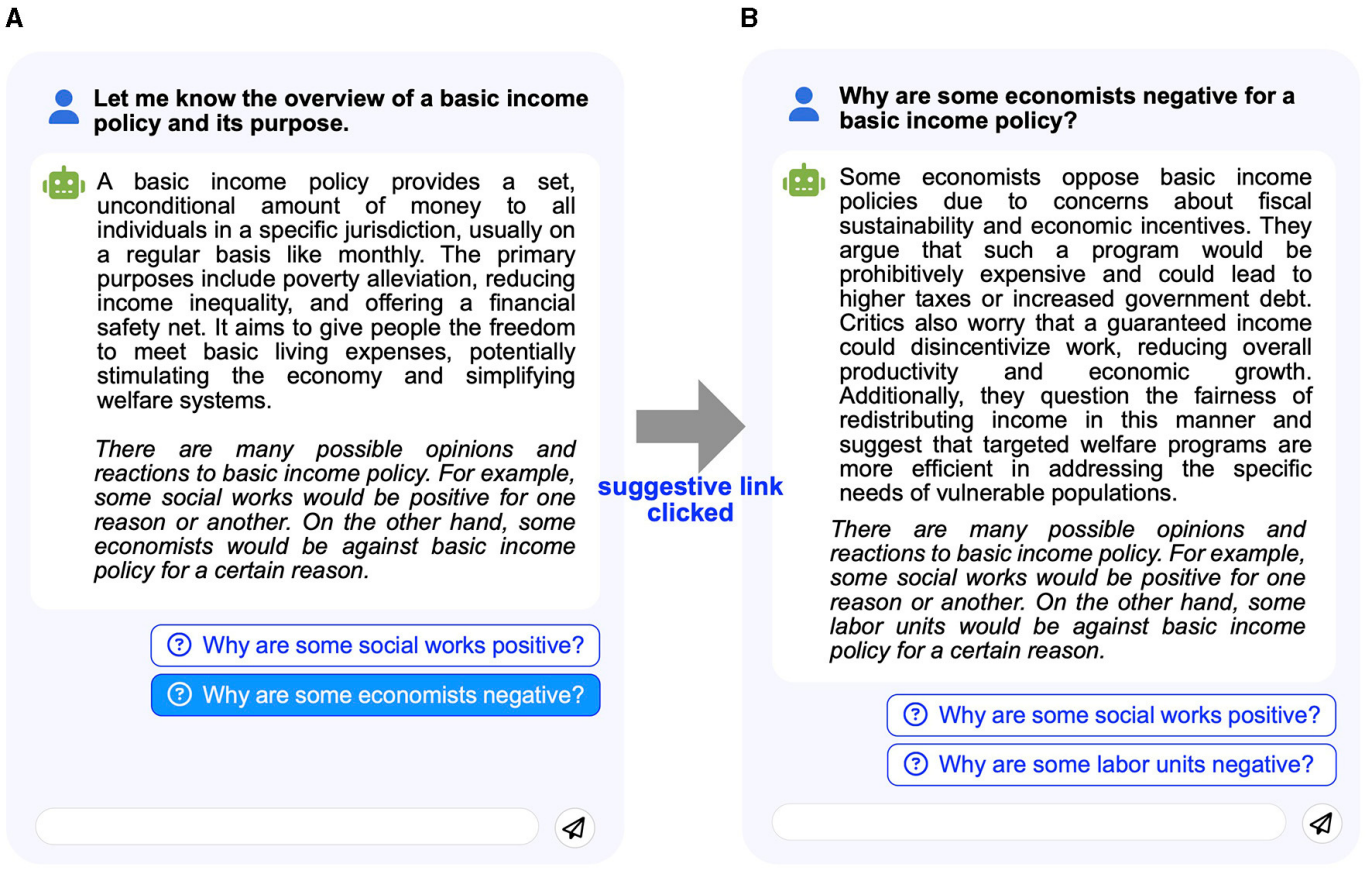
suggestive^++^ bot behavior when a participant clicks the suggestive link. **(A)** Response to initial question. **(B)** Response to suggestive click.

The suggestive^++^ bot is an extension of the suggestive bot. When providing participants with initial answers with suggestive endings, suggestive^++^ displays links to question what opinions a suggested positive/negative person might have for a given theme (referred to as *suggestive links*). Once the participants click a suggestive link to a positive/negative person, the suggestive^++^ bot displays the person's opinions against a task theme (Figure 4).

As illustrated in Figure 3B, each positive/negative person's opinion is generated via OpenAI's API using the prompt to question what opinions the person might have for the *initial answer* text. Similar to *initial answers*, the suggestive^++^ bot suggests an example of a positive and a negative person at the end of the generated opinions. In addition, the bot lists *suggestive links* to other people's opinions. In other words, once they click a *suggestive link*, the participants could see other *suggestive links*. Similar to the case of *initial answers*, we generated and cached positive/negative people's opinion texts for the task themes before the user study. We expected that the suggestive^++^ bot could encourage the participants to recall more easy-to-query questions than suggestive bot.

#### 4.2.3 Expositive bot

In addition to suggesting the existence of positive/negative people, the expositive shows one-line summaries of their opinions in the *initial answers* as supplementary information. Participants using expositive bots can briefly learn the possible perspectives or opinions of a positive and negative person without additional questioning.

The following is the procedure of *initial answer* generation in the expositive bot:

1. Similar to the suggestive bot, the expositive bot generates a plain *initial answer* and a list of positive/negative people for a given theme.
2. Similar to the suggestive^++^ bot, the expositive bot generates opinions for randomly selected positive and negative persons.
3. Each opinion is summarized in a one-line sentence via the GPT API.
4. The expositive bot puts the summarized sentences for a positive and negative person at the end of the *initial answer*.

Note that we cached summarized one-line opinions before the study, similar to *initial answers*.

#### 4.2.4 Plain bot

Plain bot is a control UI. Unlike the other three chatbots, this chatbot generates simple answers to participant queries via the GPT API. For *initial questions*, the bot displays the cached *initial answers*.

#### 4.2.5 Common setting to all UI conditions

To ensure that the OpenAI API responses for a question were not truncated every time participants issued questions to the chatbot^8^, we added a prompt-limited answer length of 400 Japanese characters (about 200 English words) for their questions. If the chatbot did not receive responses from the API within 10s, the chatbot displayed the following message: “The query has failed. Please reissue your question.” In all UI conditions, we cached the API results to new queries for stable chatbot responses to participant queries. We configured the bots to display the generated answers within 7–10s when using the cached results.

### 4.3 Procedure

First, the participants were asked to read an overview of our user study and the treatment of their collected data on a crowdsourcing website. After agreeing to a consent form, the participants were transferred to our website to start their participation in the user study. Each participant was then randomly allocated to a UI environment and three decision-making tasks. To ensure that all participants view our system information with the same layout, only PC-based log-ins were allowed (i.e., no participant could access the study if one uses a tablet or a smart phone).

Then, the participants read a description of a task flow and the chatbot used in the study. Assuming that some participants were unfamiliar with chatbot systems for IR, we made the description of our chatbot system as comprehensible as possible. Moreover, we required participants to click a “read next” button every time they read a portion of the description to ensure that they read it completely.

Next, the participants were asked to conduct a practice task to familiarize themselves with their allocated chatbot. In the practice task, the participants were asked to summarize their opinions on introducing a universal basic income system in Japan.

Afterward, the participants performed the three main tasks for the three themes randomly allocated to them from the nine themes listed in Table 1. The main task order was randomized for each participant. In each main task, the participants performed the following three subtasks for each of the allocated themes: (1) pre-questionnaire, (2) decision-making, and (3) post-questionnaire tasks.

In the pre-questionnaire task, the participants ranked their interest and knowledge of each main task theme using a seven-point Likert scale (1, not at all; 7, very much).

Subsequently, the following scenario was presented to each participant ([THEME] is a task theme):

“*Imagine the following case. The introduction of* *[THEME]* *has been discussed in your city. After the discussion in the city council, the city decided whether* *[THEME]* *is introduced or not, based on the interview with several residents. You are selected as an interviewee and need to explain whether you support* *[THEME]**. Your answer will have a substantial influence on the city policy. So, you are about to collect information about* *[THEME]* *for your decision-making by using the latest chatbot system. Collect necessary information with the chatbot. When you reach a satisfactory conclusion, summarize your opinion with reasons and fill it in on an answer form.”*

After reading the scenario, the participants were invited to start the decision-making task by clicking a dedicated button. The browser opened a webpage, where the participants interacted with the allocated chatbot and reported their opinions. At this stage, we set an initial question such as “*Let me know the overview of* *[THEME]* *and its purpose”* in the chatbot query box. Thus, all participants would ask the chatbot the initial question and collect information if necessary. The participants then reported their opinions when they reached their conclusion.

In the post-questionnaire task, we surveyed how many times our chatbot failed to generate answers during the decision-making task. These situations occurred because the chatbot occasionally failed to fetch the OpenAI GPT API within a limited time. For this survey, we asked the participants the following question: *How many times did you see the message “The query failed. Please enter your question again.”* The participants reported the error frequency on a five-point Likert scale (1, never; 2, once; 3, 2–3 times; 4, 4–5 times; 5, more than five times).

At the end of the three main tasks, we administered an exit questionnaire to obtain feedback regarding the chatbot systems. The participants also answered the daily usage of chatbot systems for IR and demographic questions related to gender, age, and education.

### 4.4 Participants

We recruited 300 participants using Lancers.jp^9^, a Japanese crowdsourcing service. Nevertheless, 18 participants were excluded from the analysis because 1 participant violated an instructional manipulation check (IMC) (Oppenheimer et al., 2009) in the exit questionnaire, 15 participants had more than one chatbot failure case in responding to their queries, and 2 participants completed the tasks without using our chatbot. Thus, only 282 participant responses were analyzed. All participants were Japanese (male = 191; female = 87; others= 4). Most participants were in their 30s and 40s (20s = 5.7%; 30s = 27.3%; 40s = 44.0%; 50s = 17.7%; others = 5.4%). Furthermore, about half of the participants reported that they never used chatbots for IR, such as ChatGPT, Google Gemini, and Bing Copilot^10^ (never used = 45.7%; once every several months = 10.3%; once a month = 13.5%; once a week = 16.3%; once every several days = 7.8%; several times a day = 6.3%). Participants were randomly assigned to one of the four UI conditions (plain = 66; expositive = 71; suggestive = 78; suggestive^++^ = 67). They used their PC or Mac to join our online user study. All participants who completed the tasks received 400 Japanese yen (approximately $2.75). On average, the participants finished all tasks within 48 min (*median*: 43 min).

### 4.5 Measurements

#### 4.5.1 Task duration

We measured the *task duration*, corresponding to the time spent on a decision-making task per theme. Task duration is often used to examine how much effort users make in learning during the information-seeking process (Câmara et al., 2021). In our study, we defined the *task duration* as the time span from the moment when chatbot interfaces were displayed to the moment when the participants reported their opinions.

During the user study, participants engage in a critical learning activity, requiring them to not only look up unfamiliar topics but also analyze the task theme and summarize their opinions from various perspectives. This type of learning is often referred to as *critical learning* (Lee et al., 2015). Within the information retrieval community, researchers often use task/search duration as a measure of critical learning engagement and effort during information-seeking activities (Yamamoto and Yamamoto, 2018; Câmara et al., 2021; Roy et al., 2021). However, studies have shown that people interacting with chatbots, like ChatGPT, tend to spend less time on search tasks compared to conventional web search engines (Xu et al., 2023). Therefore, we consider that task/search duration could be a valuable metric to assess how effectively our chatbot strategy promotes critical learning during conversational searches.

#### 4.5.2 Search frequency

We measured the *search frequency*, corresponding to the number of times the participants issued queries to the chatbots during their decision-making tasks. Similar to task/search duration, this metric is also often used to evaluate how willing people are to learn a topic in the fields of information retrieval and human-computer interaction. The *query issue count* can be regarded as how the participants came up with questions in their minds while interacting with the chatbots for their decision-making. We also measured the recommended queries (i.e., *suggestive links*) that the participants with suggestive^++^ bot clicked as well as the queries that the participants filled in the chat box by themselves.

#### 4.5.3 Opinion length

We examined how many tokens (terms) are contained in the participants' reported opinions. In the study, we asked the participants to report their opinions with reasons without setting minimum requirements for opinion length. We assumed that the more persuasive opinions the participants were encouraged to write, the longer their opinions would be. Therefore, we calculated the token-based length of participant opinions using *MeCab*, a Japanese morphological analyzer^11^.

#### 4.5.4 Perspective in opinion

We calculated the number of perspectives in the participants' opinions to investigate whether they summarized their opinions from various perspectives. This approach aligns with the concept of *T-Depth*, a metric introduced by Wilson and Wilson (2013), designed to evaluate the coverage of subtopics in participant opinions. *T-Depth* has been used in several studies to measure learning outcomes during information-seeking activities (Wilson and Wilson, 2013; Roy et al., 2021). Our indicator is a simplified version of *T-Fact*; it focuses only on the number of distinct perspectives rather than seeing how deeply participants mention each subtopic. This simplification stems from the challenge of objectively evaluating the depth of opinion on subtopics.

The themes listed in Table 1 are popular debating topics in Japan. Therefore, many books and webpages organize and list perspectives for discussion of themes. Our research group members collected and aggregated perspectives for each theme from the Web. Then, they used the list of aggregated perspectives to manually check which aspect appeared in each participant's opinion. It should be noted that the number of perspectives varied depending on the themes. Therefore, we rescaled the number of appearing perspectives in participant opinions by the expected maximum value (the number of collected perspectives per theme in Table 1).

As Sharma et al. (2024) have shown, conversational searches facilitated by LLMs often lead people to inquire about biased topics, resulting in more selective search behaviors. Therefore, we consider that the number of perspectives is a significant indicator to how effective our chatbot strategy is to promote more diverse information-seeking.

### 4.6 Statistical analyses

To examine the effect of suggestive endings in AI-powered chatbots, we analyzed the collected behavior logs and participant questionnaire responses using an analysis of covariance (ANCOVA). We conducted an ANCOVA to examine the main effect of *UI conditions* on the following measurements: (1) task duration, (2) questioning (search) frequency, (3) token length of opinions, and (4) the number of aspects in opinions. In the ANCOVA, we treated *familiarity, interest* of task themes, and *use frequency of chatbot for IR* as covariates to control personal factors. In *post hoc* tests, we conducted the Benjamini–Hochberg false discovery rate (FDR) correction (Benjamini and Hochberg, 2000) to make multiple comparisons between the UI conditions. In the ANCOVA and *post hoc* tests, we conducted log transformation for task duration, questioning frequency, and token length of opinions since the data did not follow Gaussian distributions.

## 5 Results

### 5.1 Task duration

**Figure 5:**
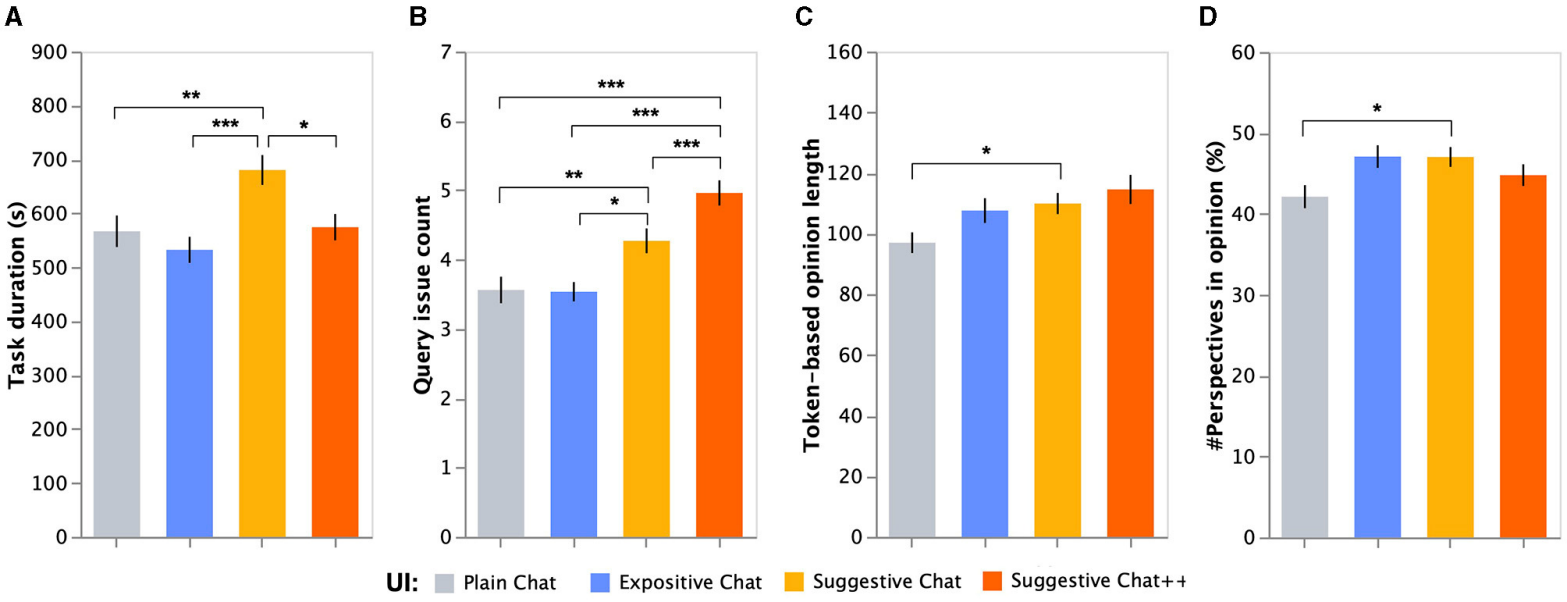
Mean and standard errors of **(A)** task duration, **(B)** search frequency, **(C)** opinion length, and **(D)** perspective count in opinion, depending on UI conditions. ***, significance level at 0.001; **, significance level at 0.01; *, significance level at 0.05, adjusted using the Benjamini–Hochberg FDR corre ction).

We investigated the time and effort invested by participants for the decision-making tasks. Figure 5A illustrates the mean and standard error of the task duration. The ANCOVA result showed a significant impact of the UI conditions on the task duration per task, after controlling individual factors (*F*_(3, 839)_ = 6.28, *p* < 0.001). Moreover, we observed a statistically significant difference between interest in themes (a covariate) on task duration (*F*_(1, 839)_ = 5.29, *p*0.05).

The *post hoc* tests revealed that the participants using the suggestive bot spent 114s longer in decision-making tasks compared to those using the plain bot (*mean*: 680.3s vs. 566.6s; *p*(*suggestive*−*plain*) < 0.001). Moreover, *post hoc* tests showed that participants using the suggestive^++^ and expositive bots completed the tasks significantly faster than those using the suggestive bot (*mean*: 574.3s vs. 532.3s vs. 680.3s; *p*(*suggestive*−*suggestive*^++^) < 0.05, *p*(*suggestive*−*expositive*) < 0.01). Nonetheless, no significant differences were observed between the suggestive^++^ and plain bots and between the expositive and plain bots.

In summary, the suggestive bot encouraged the participants to conduct a decision-making task more slowly than any other UIs. By contrast, the suggestive^++^ and expositive bots did not seem to affect the participants' behavior. These findings suggest that the participants using the suggestive bot appeared to invest more effort in collecting clues for their decision-making or organizing their opinions compared to those using the plain and suggestive^++^ bots.

### 5.2 Search frequency

We investigated how frequently the participants asked their chatbots to collect clues for the decision-making tasks. Figure 5B illustrates the mean and standard error of the search frequency. The ANCOVA result showed that the UI conditions had a significant impact on query issue count after controlling individual factors (*F*_(3, 839)_ = 17.7, *p* < 0.001). No statistically significant differences were observed between interest, knowledge of themes, and daily usage of chatbots for IR on query issue count.

The *post hoc* tests revealed that the participants significantly asked more questions to the suggestive bot compared to the plain and expositive bots (*mean*: 4.27 vs. 3.56 vs. 3.54; *p*(*suggestive*−*plain*) < 0.01, *p*(*suggestive*−*expositive*) < 0.05). Furthermore, we observed that suggestive^++^ bots encouraged participants to ask the bot more frequently compared with the suggestive, expositive, and plain bots (*mean*: 4.96 vs. 4.27 vs. 3.56 vs. 3.54; *p*(*suggestive*^++^−*suggestive*) < 0.001, *p*(*suggestive*^++^−*expositive*) < 0.001, *p*(*suggestive*^++^−*plain*) < 0.001). It should be noted that the participants using the suggestive^++^ bot queried with suggestive links at 3.55 times per task and queried without the links (querying by themselves) at 2.04 times per task on average. suggestive^++^ bot enables people to ask the bot questions just using suggestive links, whereas people using suggestive bot have to think about questions and type them in the bot by themselves. Therefore, These statistics show that participants using suggestive^++^ bot were quite willing to use the suggestive links during the tasks. The *post hoc* test results revealed that the expositive bot promoted active searches compared to the plain bot.

The above findings suggest that if the answer of the suggestive bot ended with a suggestion regarding the existence of positive/negative opinions, participants were willing to ask questions to the bot more than what they would do with the plain and expositive bots, which proactively and explicitly describe positive/negative opinions. This tendency could be stronger if the suggestive^++^ bot displayed links to issue queries for viewing detailed information on positive/negative opinions.

### 5.3 Opinion length

The length of the participant reports submitted as task answers was considered as a metric to examine the decision-making level promoted by the four chatbot types. Figure 5C illustrates the mean and standard error of the token-based opinion length. The ANCOVA result showed that the UI conditions had a significant impact on token-based opinion length after controlling individual factors (*F*_(3, 839)_ = 2.80, *p* < 0.05). We observed that two individual factors (covariates), i.e., interest in themes (*F*_(1, 839)_ = 9.21, *p* < 0.01) and knowledge on themes (*F*_(1, 839)_ = 4.62, *p* < 0.05), significantly affected the opinion length.

The *post hoc* tests revealed that the participants using the suggestive bot wrote longer opinions compared to those using the plain bot (*mean*: 109.9 tokens vs. 97.0 tokens; *p*(*suggestive*−*plain*) < 0.05). No significant difference was observed between the suggestive^++^ and plain bots (*p*>0.05), although the mean opinion length of the suggestive^++^ bot was higher than that of the suggestive bot (*mean*: 114.5 vs. 109.9). Furthermore, no significant difference was observed between the expositve and plain bots.

These results indicate that if the participants found the existence of positive/negative people for themes using suggestive bots, they were likely to explain their opinion with more words than those using the plain bot, which just answered given questions straightforwardly. In addition, the results indicate that the expositve bot did not have a large influence on opinion volume, despite providing richer answers to initial questions than the plain bot.

### 5.4 Perspectives in opinion

We investigated how many possible perspectives appeared in the participants' submitted opinions to examine if they wrote their opinions from various perspectives. Figure 5D illustrates the mean and standard error of the perspective count. The ANCOVA result revealed that the UI conditions had a significant impact on the rescaled number of perspectives in opinion after controlling individual factors (*F*_(3, 838)_ = 2.82, *p* < 0.05). No statistical significance was observed in individual factors (interest in themes, knowledge of themes, daily usage of chatbots for IR).

The *post hoc* tests showed that the participants using the suggestive bot referred to significantly more perspectives in their opinions than those using the plain bot (*mean*: 47.0% vs. 42.1% of possible perspectives; *p*(*suggestive*−*plain*) < 0.05). No significant difference was observed between the suggestive^++^ and plain bots (*p*>0.05), although the participants using the suggestive^++^ bot did more chatbot searches compared to those using the plain and suggestive bots. Moreover, no significant difference was observed between the expositive and plain bots (*p*>0.05), although the mean opinion length of the expositive bot was higher compared to that of the suggestive bot (*mean*: 47.1% vs 47.0%).

These results indicate that the participants using the suggestive bot were likely to summarize their opinions from various viewpoints compared to those using the plain bot. Furthermore, the results indicate that regardless of the richer answers provided by the expositve bot to initial questions compared to the plain bot, the participants did not formulate their opinions from multiple perspectives.

### 5.5 Qualitative analysis

We analyzed the free-form responses in the exit questionnaire to explore the participants' strategies for their decision-making. In the exit questionnaire, the participants were asked to report how they organized and summarized their opinions during the decision-making tasks. Our research group members conducted an open coding (Lewins and Silver, 2014) for the participants' reports to explore the types of participant strategies.

#### 5.5.1 Examination from various perspectives

Some participants stated that they made decisions based on various perspectives (e.g., advantages and disadvantages of a given theme). The following comments are from participants who reported that they considered various perspectives (translated from Japanese to English):

*(P19 with suggestive bot) “I was careful not to favor one side over the other by making the chatbot present information on both pros and cons. I also verified my prior knowledge, comparing the chatbot responses with my own views.”*

*(P47 with expositive bot) “To write solid opinions, I collected information from two perspectives: pros/cons and positive/negative opinions.”*

Meanwhile, the following comment is from a participant who was thought not to consider various perspectives:

*(P11 with plain bot) “After deciding my stance, either for or against a given theme, I used the chatbot to collect information supporting my stance.”*

We examined the ratio of participants who clearly commented that they considered various perspectives during the tasks depending on the UI conditions. The ratios were 52.1%, 34.9%, 62.8%, and 62.7% for the participants using the expositive bot, the plain bot, the suggestive bot, and the suggestive^++^ bot, respectively. The χ^2^ tests with the Bonferroni adjustment revealed that the ratios of the suggestive and suggestive^++^ bots were significantly higher than that of the plain bot (*p*(*suggestive*−*plain*) < 0.05/6; *p*(*suggestive*^++^−*plain*) < 0.05/6). These results indicate that if the chatbots implicitly suggested the existence of positive/negative opinions, the participants could be more careful about various perspectives in their decision-making. By contrast, even if the expositve bot complemented a brief summary about a positive and a negative person's opinion to initial answers, the participants did not try to make their decisions from multiple perspectives.

#### 5.5.2 How to use chatbots

Different participants used the chatbots for different reasons. Some participants used the chatbots to learn about unknown concepts from the chatbot's answers, as represented by the following comments:

*(P26 with expositive bot) “I read the chatbot's answer. Then I queried the chatbot to summarize my answer if I came up with questions.”*

*(P229 with plain bot) “I asked the bot about what I was curious about or did not understand and then summarized my opinion.”*

Other participants used the bots to collect clues for their decision-making. Some participants thoughtfully considered various perspectives or weighed the pros and cons of the given themes to inform their decisions as follows:

*(P59 with suggestive bot) “I compared opinions from both supporters and opponents. Then I organized those opinions closer to my own thinking.”*

*(P170 with suggestive*^++^ *bot) “I made sure to check both positive and negative opinions before forming my own view. I queried the chatbot about positive opinions and negative opinions by turns.”*

Some participants also tried to corroborate their opinions (prior beliefs) with the chatbots to gather supportive data and expected counterarguments such as the following:

*(P102 with suggestive*^++^ *bot) “I started by reviewing the provided theme overview and determined my stance. I then searched for supportive reasons and opposing ones and selected persuasive arguments to consolidate my own opinion. If I didn't find a decisive reason in the first search, I conducted a further, more in-depth survey using the bot.”*

*(P148 with suggestive bot) “Firstly, I received an overview of the theme and then inquired about the details of opposing opinions. After that, I formulated my arguments, constructing a rebuttal.”*

As already described, the participants' comments in the exit questionnaire indicate that the suggestive and suggestive^++^ bots promoted the participants' awareness of decision-making from both positive and negative perspectives on the given themes. The following comments indicate that participants thought suggestive links provided by the suggestive^++^ bot are useful in searching for detailed information regarding positive/negative perspectives efficiently:

*(P6 with suggestive*^++^ *bot) “Once I understood the overview, the task was not so difficult. I didn't come up with new questions by myself, so I relied more on suggestive links rather than manually asking the bot questions.”*

*(P281 with suggestive*^++^ *bot) “The system allowed me to choose between pro and con opinions (links) on the theme. I used this feature to validate my own views.”*

However, the way of using suggestive links depended on the participants. Some participants using the suggestive^++^ bot implied that they used either links to positive opinions or links to negative opinions as follows:

*(P87 with suggestive*^++^ *bot) “I used the system to investigate what risks might be involved. If the risks (negative opinions) on a theme were low, I tried to have positive opinions on it.”*

*(P241 with suggestive*^++^ *bot) “Firstly, I determined whether I was in favor or against the given theme, and then I used the chatbot to search for data supporting my view.”*

The suggestive bot did not provide suggestive links compared with the suggestive^++^ bot; however, it prompted the participants to ask about or reflect on positive/negative people's opinions on the theme:

*(P203 with suggestive bot) “In answer to the initial question, the bot showed a description suggesting that I should seek further clarification on positive and negative opinions, so I started by following the suggestion.”*

*(P280 with suggestive bot) “Firstly, I was curious about what the pros and cons might be, so I searched for those aspects. While considering the pros and cons of the theme, I checked current statistics or data to ensure that I tried to form a fair opinion.”*

As the below comment suggests, some participants using the expositive bot would not be willing to ask questions as they would feel that the bot provided sufficient information for their decision-making:

*(P277 with expositive bot) “Most information from the bot was usable as-is, so I actively used them.”*

#### 5.5.3 Complaints

A few participants complained that the chatbot's answers sometimes seemed wrong or unreliable, thereby hoping that the bots could provide more detailed information and evidence. Moreover, a few participants complained that the chatbot's information was difficult to read.

## 6 Discussion

After controlling the individual factors, our study results revealed that the suggestive and suggestive^++^ bots significantly influence the participants' behavior and attitude in their decision-making tasks.

As for **RQ1**, the suggestive bot caused the participants to spend the longest time in decision-making tasks among the four UI conditions. Moreover, the suggestive bot promoted more frequent information seeking compared to the plain and expositive bots. It also encouraged the participants to write longer texts regarding their opinions compared to those using the plain bot. Therefore, we conclude that the suggestive bot can encourage users to put more effort into formulating their opinions and gathering information for decision-making from time and content perspectives.

As for **RQ2**, our qualitative analysis revealed that more participants using the suggestive and suggestive^++^ bots were aware of both the pros/cons perspectives in their decision-making compared to those using the plain bot. Furthermore, our behavior analysis showed that the participants using the suggestive bot were likely to refer to more perspectives in their opinion reports compared to those using the plain bot, whereas the suggestive^++^ did not indicate such a tendency. We conclude that the suggestive bot can encourage users to formulate their decision from various viewpoints.

As for **RQ3**, the suggestive^++^ bot, providing links to survey positive/negative people's opinions along with the suggestive answers, promoted more frequent search activities compared to any other UI. In addition, the suggestive^++^ bot significantly reduced the time cost for the tasks compared to the suggestive bot. In the exit questionnaire, 62.7% of participants using the suggestive^++^ bot reported that they tried to formulate their opinions as objectively as possible from both sides of pros and cons. However, the behavior analysis result showed that the suggestive^++^ bot did not encourage participants to report long opinions with various perspectives compared to the suggestive bot. These results indicate that the suggestive^++^ bot did not substantially promote critical decision-making activities, although it could improve information-seeking efficiency. We believe that such noncritical behaviors can be attributed to the cognitive bias in information seeking (White, 2013; Azzopardi, 2021), such as *selective exposure* (Liao et al., 2015) and *confirmation bias* (Kahneman, 2011; Suzuki and Yamamoto, 2021). The comments of P87 and P241 in the qualitative analysis suggest the influence of selective exposure and confirmation bias on the users' behaviors. However, it is worth noting that our interpretations above are based only on the submitted task reports and the participants' reflective comments in the exit questionnaire. To ensure whether the suggestive^++^ bot can promote critical decision-making, a further study of the cognitive process during decision-making tasks with the chatbots should be conducted via laboratory experiments.

As for the expositive bot, we found no significant effects compared to the plain bot. When querying a theme overview at the beginning of the tasks, the participants using the expositive bot saw a brief summary of a positive/negative person's opinions without additional queries. In other words, the bot explicitly complemented short, two-sided information for task themes, although the complemented information is not sufficient to make critical judgments on the task themes. However, as P277 suggested, the expositive bot creates the impression of providing sufficient information. This drives participants to pick up only their favorable information to summarize their opinions. Therefore, even if the participants used the expositve bot, they would not exert much effort toward critical decision-making.

As for **RQ4**, we confirmed that the knowledge of themes affected time efforts in decision-making tasks, while the interest in themes affected the length of reported opinions. These results indicate that knowledge of and interest in themes could affect the amount of effort in decision-making with AI-powered chatbots.

In the end, we conclude that *suggestive endings*, which hint at something in chatbot interaction, can draw more spontaneous questions from users and encourage them to formulate their opinions from various perspectives rather than provide definitive answers or predefined questions (such as in the suggestive++ bot).

## 7 Limitations and potential challenges

Our study showed that the suggestive ending strategy in a human–chatbot interaction can be useful in enhancing critical decision-making. However, the study has some limitations and several challenges still exist toward better AI-based decision-making support.

One limitation is an experimental environment. We used a crowdsourcing platform for our user study. Although user studies with crowdsourcing have been more popular, this approach has several concerns, including the demographic biases of crowd workers, the presence of lazy participants, and the control of experimental environments (task times and devices for experiments) (Ross et al., 2010). As a result, our study's participant pool might not accurately reflect the general population, and some participants might not have performed the tasks seriously.

Another limitation the display timing of suggestive endings. In the study, the suggestive bot provided answers with suggestive endings only for the initial questions. Therefore, we need to investigate the effects depending on the timing and context of suggestive ending presentations. Moreover, we relied only on the analysis of participants' behaviors during the main tasks and their comments in the exit questionnaire to understand their strategy for decision-making. Think-aloud protocols and stimulated recalls should be conducted in laboratory experiment settings to understand the cognitive decision-making process with chatbots better.

A possible challenge is the topic on which chatbot hints. In the study, we focused on suggesting who is positive or negative for a theme, aiming to make participants aware of the pros/cons viewpoints and to draw spontaneous questions to foster their understanding of the theme (e.g., “*[Occupation name] people can be positive for [THEME] with a certain reason”*). However, other factors can affect critical decision-making and information seeking. For example, researchers in information and media literacy have stated that currency, relevance, authority, accuracy, and purpose are important to check for critical judgment on the quality of claims and information (Musgrove et al., 2018). Therefore, the chatbots should determine a focused factor and create associated suggestive endings depending on the context of decision-making. For example, if users are encouraged to explore various information from the currency viewpoints, a possible suggestive ending can be “*The above opinion was mainstream in the 2010s, but completely different opinions are prevalent in the 2020s”*. A remaining issue is a method to automatically generate effective suggestive endings.

The second challenge is related to the proper use of chat strategies to enhance cognitive activities. In this study, we focused on hinting at something in chatbot answers to draw spontaneous questions from users. However, there can be other ways to draw cognitive efforts toward critical decision-making, such as AI questioning and forcible time setting for thinking (Buçinca et al., 2021). As for the AI-questioning approach, devising what and how to make chatbots ask would enable them to promote various cognitive activities, such as logical reasoning (Danry et al., 2023) and reflecting on lacking issues of one's view (Okuse and Yamamoto, 2023). Nevertheless, explicit questioning might make users intrusive and uncomfortable depending on the frequency, timing, or user personality. Furthermore, even if chatbots provide questions and suggestive endings for users, some users may have difficulties in finding answers and related information by themselves (Odijk et al., 2015). Therefore, the chatbots for decision-making support should use explicit questioning (instructive intervention), suggestive endings in answers (modest intervention), and detailed explanations, depending on the situation and users' personal factors. Moreover, the chatbots should encourage users to perform Web searches without an overreliance on the bots as necessary so that users can corroborate their opinions and the bot's answers from various sources.

## 8 Conclusion

Although people use generative AIs with LLMs to readily obtain information relevant to their requirements, their overreliance on AIs can cause shortsighted decision-making and weaken cognitive skills. Our proposed suggestive chatbot encourages people to have spontaneous questions for critical decision-making on a given theme by ending an answer that hints at potentially interest-triggering points.

The online user study revealed that the suggestive bot encouraged participants to exert more effort in developing their opinions and gathering information for decision-making compared with simple chatbots. Moreover, the study showed that the suggestive bot encouraged participants to make their decisions from various perspectives. We did not observe such a tendency with the expositve chatbot, which complemented information from a specific perspective. These findings indicate that AI-powered chatbots can better enhance human decision-making with *suggestive endings*, which leave room for questions and discussions rather than definitive explanations to a question (query).

Our proposed method has several challenges for improvement. These include investigation on how to use suggestive endings, questioning, and definitive explanations depending on situations and laboratory studies to understand the cognitive processes during decision-making tasks using our chatbot strategy. However, we believe that *suggestive endings* in chatbot answers constitute a good strategy for AI-powered chatbots to enhance critical information seeking and decision-making.

## Data availability statement

The raw data supporting the conclusions of this article will be made available by the authors, without undue reservation.

## Ethics statement

Ethical review and approval was not required for the study on human participants in accordance with the local legislation and institutional requirements. The patients/participants provided their written informed consent to participate in this study. Written informed consent was obtained from the individual(s) for the publication of any potentially identifiable images or data included in this article.

## Author contributions

YY: Conceptualization, Formal Analysis, Methodology, Software, Writing – original draft, Writing – review & editing.

## Footnotes

^1^The Scholastic Assessment Test (SAT) is a standardized test for college admissions across the United States. It is designed to assess students' proficiency in mathematics, reading, and writing.

^2^ https://www.nytimes.com/2023/03/14/technology/openai-new-gpt4.html

^3^ChatGPT: https://chat.openai.com/.

^4^Google Gemini: https://gemini.google.com/.

^5^Amazon Mechanical Turk: https://www.mturk.com/.

^6^ https://www.prolific.com/

^7^Azure OpenAI Service: https://azure.microsoft.com/en-us/products/ai-services/openai-service-b.

^8^The prompt was set invisible to the participants.

^9^ https://www.lancers.jp/

^10^Bing Copilot: https://www.microsoft.com/en-us/bing.

^11^MeCab: https://taku910.github.io/mecab/ (in Japanese).
